# Case Report: Herpes zoster-associated abdominal wall bulge with ipsilateral muscle atrophy

**DOI:** 10.3389/fmed.2026.1752137

**Published:** 2026-02-04

**Authors:** Yuxuan Wang, Juping Chen

**Affiliations:** Department of Dermatology, Affiliated Hospital of Yangzhou University, Yangzhou University, Yangzhou, China

**Keywords:** abdominal pseudohernia, abdominal wall muscle atrophy, case report, computed tomography, herpes zoster, segmental zoster paresis

## Abstract

Herpes zoster (HZ) is the reactivation of the varicella-zoster virus and usually causes unilateral dermatomal vesicles; deep tissue involvement is uncommon. We report a 76-year-old man who, 12 days after classic right-flank HZ in the T11 dermatome, developed a painless right-sided abdominal bulge. Examination found a soft, non-pitting swelling. Computed tomography demonstrated ipsilateral thinning of the abdominal wall muscles without evidence of a fascial defect, true hernia, or mass, consistent with zoster-related segmental motor neuropathy presenting as an abdominal pseudohernia. He was managed conservatively with reassurance; no surgery was required. This case highlights a rare neuromuscular complication of HZ and supports the use of imaging in unexplained post-zoster abdominal swelling.

## 1 Introduction

Herpes zoster (HZ) is the reactivation of the varicella-zoster virus, typically presenting as unilateral vesicular eruptions along a dermatome (1). Beyond its cutaneous manifestations, deeper tissue involvement is rare, with occasional reports of motor neuropathy and abdominal wall weakness (2). Herpes zoster-related motor involvement can present as an abdominal wall bulge (abdominal pseudohernia), which may be mistaken for a true hernia. We report an elderly male patient who developed a painless unilateral abdominal wall bulge shortly after thoracic HZ.

## 2 Case description

**Figure 1:**
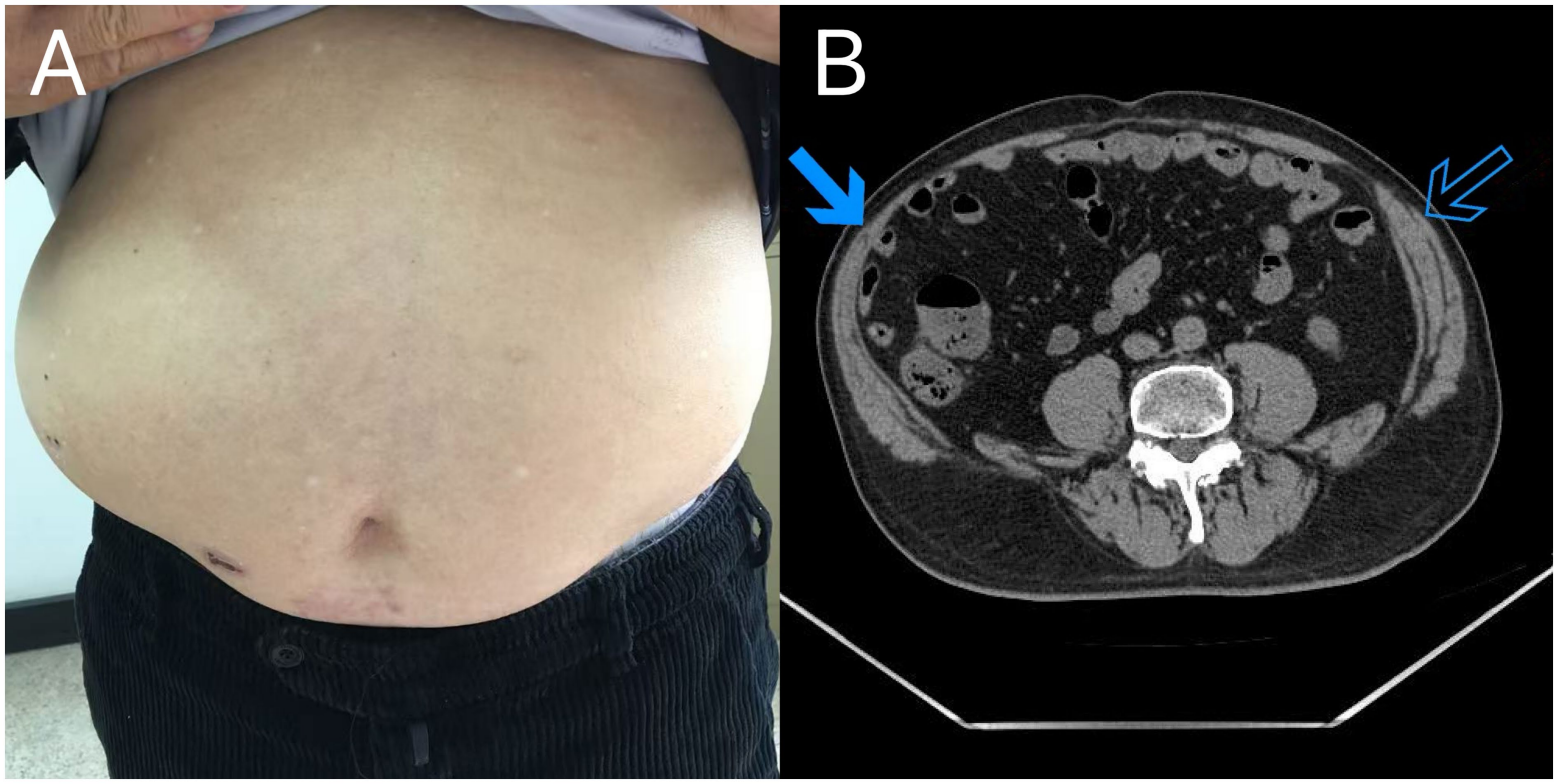
**(A)** Clinical photograph shows a right-sided abdominal wall bulge. The overlying crusts represent resolving herpes zoster vesicular lesions located in the same anatomical region. **(B)** Axial computed tomography image demonstrates ipsilateral thinning of the abdominal wall muscles on the affected side (solid arrow) compared with the contralateral side (open arrow), with no evidence of a fascial defect, true hernia, or other structural abdominal pathology.

A 76-year-old man attended the dermatology outpatient clinic with a 5-day history of pain and grouped blisters on his right flank, consistent with the T11 dermatome. He had no known underlying diseases, took no regular medications, and had no history suggestive of immunocompromise; family and psychosocial history were non-contributory. He was diagnosed with HZ and zoster-associated pain. He was started on oral famciclovir (250 mg three times daily), pregabalin (75 mg twice daily), and mecobalamin (0.5 mg three times daily), along with standard topical and supportive measures. Nine days after the first visit, he returned with new right-sided abdominal swelling of 2 days’ duration, without pain, fever, or gastrointestinal symptoms. He denied constipation and reported no change in bowel habits. The rash had crusted, and examination revealed a soft, non-pitting bulge in the lower right abdomen (Figure 1A). The location of the abdominal bulge anatomically corresponded to the previously affected T11 dermatome. CT imaging demonstrated ipsilateral thinning of the right paraspinal and lateral abdominal wall muscles compared with the contralateral side, without evidence of a fascial defect, true hernia, or other structural abdominal pathology (Figure 1B). The presentation was considered an abdominal pseudohernia secondary to zoster-related segmental motor paresis, supported by ipsilateral abdominal wall muscle thinning on CT and the absence of a fascial defect or true hernia. The patient was reassured and managed conservatively, with no surgical intervention required. Key events are summarized in Table 1.

**Table 1:** Timeline of the clinical course.

Time point	Clinical event
Day 0	Onset of right-flank vesicular rash (T11 dermatome)
Day 5	First clinic visit; diagnosed with herpes zoster and zoster-associated pain; oral antiviral and symptomatic treatment initiated
Day 12	Onset of painless right-sided abdominal bulge
Day 14	Second clinic visit; CT imaging shows ipsilateral abdominal wall muscle thinning without evidence of a true hernia

### 2.1 Diagnostic assessment

CT findings supported a diagnosis of an abdominal pseudohernia secondary to zoster-related segmental motor paresis, with no evidence of a fascial defect, true hernia, or mass. Alternative causes of abdominal wall bulging, including prior abdominal surgery, traumatic abdominal wall injury, and focal abdominal wall neuropathy unrelated to herpes zoster, were considered clinically but were not supported by the patient’s history or physical examination. No additional targeted investigations for alternative etiologies were performed.

### 2.2 Therapeutic intervention

No specific therapeutic intervention was initiated for the abdominal pseudohernia. After cross-sectional imaging excluded a true hernia or other structural abdominal pathology, the patient was managed conservatively with reassurance, explanation of the benign and self-limited nature of the condition, activity guidance, and scheduled follow-up. No surgical or additional medical treatment was required.

### 2.3 Follow-up and outcomes

At the 1-month follow-up after rash onset, the right-sided abdominal bulge persisted without clear improvement. The patient continued to experience postherpetic neuralgia, which had improved compared with baseline but had not fully resolved. He reported no constipation or change in bowel habits and developed no additional neurological symptoms. Medication adherence was good, and no adverse effects were reported. No repeat imaging or electrophysiological testing was performed during the follow-up because the clinical course remained stable and there were no red-flag symptoms. No surgical intervention was required.

### 2.4 Patient perspective

The patient reported initial concern about the sudden abdominal bulge but felt reassured after imaging demonstrated no hernia. He appreciated conservative management and regular follow-up.

## 3 Discussion

Motor involvement is a rare complication of herpes zoster, reported in approximately 1–5% of cases, and may manifest as segmental zoster paresis. Segmental zoster paresis has been estimated to occur in approximately 0.5–1% of all herpes zoster infections. When thoracoabdominal myotomes are affected, the resulting abdominal bulge due to abdominal wall weakness can mimic a true abdominal hernia and is commonly described as an abdominal pseudohernia; this presentation appears to be exceedingly uncommon, with only several dozen cases reported in the literature, suggesting that it is far less common than segmental zoster paresis overall (3, 4). In this setting, cross-sectional imaging is particularly useful to exclude a fascial defect, true herniation, or other structural abdominal pathology, which can help prevent unnecessary surgical intervention.

The pathophysiology of segmental zoster paresis is not fully established. A proposed explanation is the extension of VZV-associated inflammation from sensory structures (e.g., dorsal root ganglia) to motor pathways, such as the anterior roots and/or anterior horn, leading to focal weakness in the same or adjacent segments as the rash (5). In our patient, the temporal and segmental concordance between the T11 herpes zoster eruption and the subsequent unilateral abdominal bulge, together with CT evidence of ipsilateral abdominal wall muscle thinning, supports zoster-related motor involvement presenting as an abdominal pseudohernia.

When evaluating unilateral abdominal wall bulging, a differential diagnosis should be considered, including structural causes such as a true abdominal wall hernia, fascial defects, or focal lesions; neurogenic causes unrelated to herpes zoster, including diabetic truncal neuropathy, thoracolumbar radiculopathy, or post-surgical denervation; and neoplastic causes, such as primary or metastatic soft tissue tumors of the abdominal wall (6).

In the present case, cross-sectional imaging demonstrated intact fascia without a focal mass or defect, making structural and neoplastic etiologies unlikely. Neurogenic causes other than herpes zoster were also considered less likely given the absence of diabetes, prior surgery, trauma, or spinal symptoms.

With regard to treatment, although early antiviral therapy is well established for general herpes zoster outcomes, its specific effect on motor complications remains uncertain (7, 8). Available evidence for segmental zoster paresis/abdominal pseudohernia is largely limited to case reports and small retrospective series, with no prospective controlled data confirming a benefit from earlier initiation (9, 10).

Electrophysiological studies can demonstrate denervation in affected muscles and may help confirm abdominal wall involvement, although EMG was not performed in the present case. Clinically, the onset of paresis has been reported from several days to weeks after eruption, often approximately 2 weeks; our patient developed abdominal bulging 12 days after the rash, which falls within the reported timeframe (5). Gastrointestinal complications such as ileus or colonic pseudo-obstruction have been described in some cases, underscoring the importance of assessing bowel symptoms; notably, our patient denied constipation and reported no change in bowel habits (5, 11). At 1-month follow-up, the abdominal bulge persisted without additional symptoms, and no surgical intervention was required.

This report is limited by its single-case nature, the absence of electrophysiological confirmation, and limited follow-up. Nevertheless, it highlights an under-recognized motor complication of herpes zoster and emphasizes a practical diagnostic approach—recognizing abdominal pseudohernia and using imaging to exclude true hernia—to avoid unnecessary surgical referral.

## Data Availability

The original contributions presented in the study are included in the article/supplementary material, further inquiries can be directed to the corresponding author.
